# Amplitude- and Fluctuation-Based Dispersion Entropy

**DOI:** 10.3390/e20030210

**Published:** 2018-03-20

**Authors:** Hamed Azami, Javier Escudero

**Affiliations:** School of Engineering, Institute for Digital Communications, University of Edinburgh, Edinburgh EH9 3FB, UK

**Keywords:** nonlinear analysis, permutation entropy, dispersion entropy, fluctuation-based dispersion entropy, forbidden patterns

## Abstract

Dispersion entropy (DispEn) is a recently introduced entropy metric to quantify the uncertainty of time series. It is fast and, so far, it has demonstrated very good performance in the characterisation of time series. It includes a mapping step, but the effect of different mappings has not been studied yet. Here, we investigate the effect of linear and nonlinear mapping approaches in DispEn. We also inspect the sensitivity of different parameters of DispEn to noise. Moreover, we develop fluctuation-based DispEn (FDispEn) as a measure to deal with only the fluctuations of time series. Furthermore, the original and fluctuation-based forbidden dispersion patterns are introduced to discriminate deterministic from stochastic time series. Finally, we compare the performance of DispEn, FDispEn, permutation entropy, sample entropy, and Lempel–Ziv complexity on two physiological datasets. The results show that DispEn is the most consistent technique to distinguish various dynamics of the biomedical signals. Due to their advantages over existing entropy methods, DispEn and FDispEn are expected to be broadly used for the characterization of a wide variety of real-world time series. The MATLAB codes used in this paper are freely available at http://dx.doi.org/10.7488/ds/2326.

## 1. Introduction

Searching for patterns in signals and images is a fundamental problem and has a long history [1]. A pattern denotes an ordered set of numbers, shapes, or other mathematical objects, arranged based on a rule. Elements of a given set are usually arranged by the concepts of permutation and combination [2]. Combination means a way of selecting elements or objects of a given set in which the order of selection does not matter. However, the order of objects is usually a crucial characteristic of a pattern [1,2]. In contrast, the concept of permutation pattern indicates an arrangement of the distinct elements or objects of a given set into some sequences or orders [2,3,4,5]. Permutation patterns have been studied occasionally, often implicitly, for over a century, although this area has grown significantly in the last three decades [6].

However, the concept of permutation pattern does not consider repetition. Repetition is an unavoidable phenomenon in digitized signals. Furthermore, permutation considers only the order of amplitude values and so some information regarding the amplitudes may be ignored [7,8]. To deal with these issues, we have recently introduced dispersion patterns, taking into account repetitions [9].

The probability of occurrence of each potential dispersion or permutation pattern plays a key role in defining the entropy of signals [9,10,11]. Entropy is a powerful measure to quantify the uncertainty of time series [9,11]. Assume we have a probability distribution s with N potential patterns $\left\{s_{1},s_{2},\ldots ,s_{N}\right\}$. Based on Shannon’s definition, the entropy of the distribution **s** is $-\sum_{k=1}^{N}Pr\left\{s_{k}\right\}\log\left(Pr\left\{s_{k}\right\}\right)$, where $Pr\{s_{k}\}$ is the probability of occurrence of pattern $s_{k}$ [11]. When all the probability values are equal, the maximum entropy occurs, while if one probability is certain and the others are impossible, minimum entropy is achieved [9,11].

Over the past three decades, a number of entropy methods have been introduced based on Shannon entropy (ShEn) and conditional entropy (ConEn), respectively denoting the amount of information and the rate of information production [9,12,13,14]. The widely-used sample entropy (SampEn) [14] is based on ConEn [14], whereas popular permutation entropy (PerEn) and newly developed dispersion entropy (DispEn) [9] are based on ShEn [10] (we compare these methods and also evaluate the relationship between the parameters of DispEn and SampEn in Section 6).

SampEn denotes the negative natural logarithm of the conditional probability that two series similar for *m* sample points remain similar at the next sample, where self-matches are not considered in calculating the probability [14]. For detailed information, please refer to [14]. SampEn leads to undefined or unreliable entropy values for short time series and is not fast enough for long signals [15,16].

PerEn, which is based on the permutation patterns or order relations among amplitudes of a time series, is a widely-used entropy method [10]. For detailed information about the algorithm of PerEn, please see [10]. PerEn is conceptually simple and computationally quick. Nevertheless, it has three main problems directly derived from the fact that it considers permutation patterns. First, the original PerEn assumes a signal has a continuous distribution, therefore equal values are rare and can be ignored by ranking them based on the order of their emergence. However, while dealing with digitized signals with coarse quantization levels, it may not be appropriate to simply ignore them [17,18]. Second, when a time series is symbolized based on the permutation patterns (Bandt-Pompe procedure), only the order of amplitude values is taken into account and some information with regard to the amplitudes may be ignored [8]. Third, it is sensitive to noise (for further information, please see Section 6).

To deal with the aforementioned shortcomings of PerEn and SampEn at the same time, we have very recently developed DispEn based on symbolic dynamics or patterns (here, dispersion patterns) and Shannon entropy to quantify the uncertainty of time series [9]. The concept of symbolic dynamics arises from a coarse-graining of the measurements, that is, the data are transformed into a new signal with only a few different elements. Thus, the study of the dynamics of time series is simplified to a distribution of symbol sequences. Although some of detailed information may be lost, some of the invariant, robust properties of the dynamics may be kept [19,20,21]. Of note is that since the original DispEn is based on the amplitude-based symbols of signals [9], it might also be referred to as amplitude-based DispEn. Nevertheless, we will only use the term DispEn for conciseness.

The results showed that DispEn, unlike PerEn, is sensitive to change in simultaneous frequency and amplitude values and bandwidth of time series and that DispEn outperformed PerEn in terms of discrimination of diverse biomedical and mechanical states [9]. As DispEn needs to neither sort the amplitude values of each embedding vector nor calculate every distance between any two composite delay vectors with embedding dimensions *m* and m+1, it is fast [9]. The good performance of DispEn to distinguish different dynamics of real-time series was also shown in [22,23,24].

In this article, we investigate the effect of different parameters and mapping algorithms on the ability of DispEn to quantify the uncertainty of signals for the first time. Note that these issues were not the scope of our last paper, which developed DispEn [9]. Furthermore, herein, we also develop for the first time fluctuation-based DispEn (FDispEn) taking into account the fluctuations of signals. FDispEn is based on Shannon entropy and the differences between adjacent elements of dispersion patterns, named fluctuation-based dispersion patterns. We also introduce the concepts of forbidden amplitude- and fluctuation-based dispersion patterns and show that they can be used to distinguish deterministic from stochastic time series. Additionally, we compare both DispEn and FDispEn with commonly used metrics (SampEn, PerEn, and Lempel–Ziv complexity) in the analysis of two real-world datasets.

## 2. Methods

In this section, we describe DispEn and FDispEn in detail.

### 2.1. Dispersion Entropy (DispEn) with Different Mapping Techniques

Given a univariate signal $x=\{x_{1},x_{2},\ldots ,x_{N}\}$ with length *N*, the DispEn algorithm is as follows:

(1) First, $x_{j}\left(j=1,2,\;\ldots \;,N\right)$ are mapped to c classes with integer indices from 1 to c. The classified signal is $u_{j}\left(j=1,2,\;\ldots \;,N\right)$. A number of linear and nonlinear mapping techniques, introduced in Section 2.3, can be used in this step.
(2) Time series $u_{i}^{m,c}$ are made with embedding dimension *m* and time delay *d* according to $u_{i}^{m,c}=\left\{u_{i}^{c},u_{i+d}^{c},\;\ldots \;,u_{i+(m-1)d}^{c}\right\}$, i=1,2,…,N−(m−1)d [9,10]. Each time series $u_{i}^{m,c}$ is mapped to a dispersion pattern $\pi_{v_{0}v_{1}\;\ldots \;v_{m-1}}$, where $u_{i}^{c}=v_{0}$, $u_{i+d}^{c}=v_{1}$,..., $u_{i+(m-1)d}^{c}=v_{m-1}$. The number of possible dispersion patterns assigned to each vector $u_{i}^{m,c}$ is equal to $c^{m}$, since the signal $u_{i}^{m,c}$ has *m* elements and each can be one of the integers from 1 to *c* [9].
(3) For each of $c^{m}$ potential dispersion patterns $\pi_{v_{0}\;\ldots \;v_{m-1}}$, relative frequency is obtained as follows:
  (1) p(πv0…vm−1)=#{ii≤N−(m−1)d,uim,chastypeπv0…vm−1}N−(m−1)d,
  where # means cardinality. In fact, $p(\pi_{v_{0}\ldots v_{m-1}})$ shows the number of dispersion patterns of $\pi_{v_{0}\;\ldots \;v_{m-1}}$ that is assigned to $u_{i}^{m,c}$, divided by the total number of embedded signals with embedding dimension *m*.
(4) Finally, based on the Shannon’s definition of entropy, the DispEn value is calculated as follows:
  (2) $$\mathrm{DispEn}\left(x,m,c,d\right)=-\sum_{\pi =1}^{c^{m}}p(\pi_{v_{0}\ldots v_{m-1}})\cdot \ln\left(p(\pi_{v_{0}\ldots v_{m-1}})\right).$$

**Figure 1:**
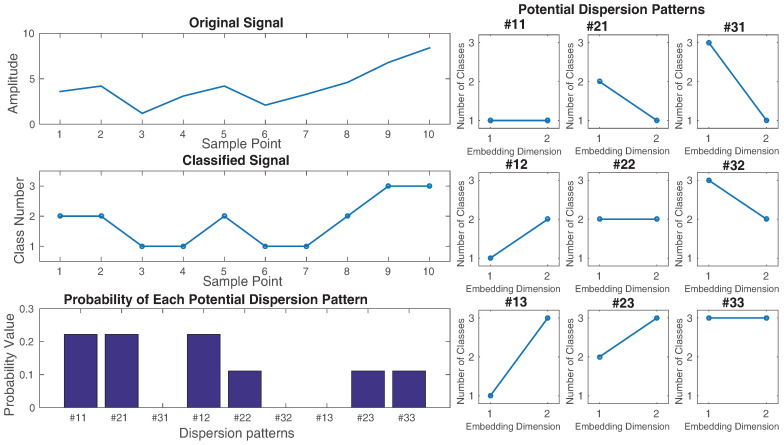
Illustration of the DispEn algorithm using linear mapping of x={3.6,4.2,1.2,3.1,4.2,2.1,3.3,4.6,6.8,8.4} with the number of classes 3 and embedding dimension 2.

As an example, let us have a series x={3.6,4.2,1.2,3.1,4.2,2.1,3.3,4.6,6.8,8.4}, shown on the top left of Figure 1. We want to calculate the DispEn value of **x**. For simplicity, we set d=1, m=2, and c=3. The $3^{2}=9$ potential dispersion patterns are depicted on the right of Figure 1. $x_{j}$ (j=1,2,…,10) are linearly mapped into three classes with integer indices from 1 to 3, as can be seen in Figure 1. Next, a window with length 2 (embedding dimension) moves along the signal and the number of each of the dispersion patterns is counted. The relative frequency is shown on the bottom left of Figure 1. Finally, using Equation (2), the DispEn value of **x** is equal to $-(\frac{2}{9}\ln\left(\frac{2}{9}\right)+\frac{2}{9}\ln\left(\frac{2}{9}\right)+\frac{2}{9}\ln\left(\frac{2}{9}\right)+\frac{1}{9}\ln\left(\frac{1}{9}\right)+\frac{1}{9}\ln\left(\frac{1}{9}\right)+\frac{1}{9}\ln\left(\frac{1}{9}\right))=1.7351$.

If all possible dispersion patterns have equal probability value, the DispEn reaches its highest value, which has a value of $\ln(c^{m})$. In contrast, when there is only one $p(\pi_{v_{0}\ldots v_{m-1}})$ different from zero, which demonstrates a completely certain/regular time series, the smallest value of DispEn is obtained [9]. Note that we use the normalized DispEn as $\frac{\mathrm{DispEn}}{\ln(c^{m})}$ in this study [9].

### 2.2. Fluctuation-Based Dispersion Entropy (FDispEn)

In some applications (e.g., in computing the correlation function and in spectral analysis), the (local or global) trends from the data [25,26] need to be removed. In these kinds of algorithms, after detrending the local or global trends of a signal, the fluctuations are evaluated [25,26]. For example, in the popular detrended fluctuation analysis technique, the local trends of a signal are first removed [27].

When only the fluctuations of a signal are relevant or local trends of a time series are irrelevant [25,26,27], there is no difference between dispersion patterns {1,3,4} and {2,4,5} or {1,1,1} and {3,3,3}. That is, the fluctuations of {1,3,4} and {2,4,5} or {1,1,1} and {3,3,3} are equal. Accordingly, we introduce FDispEn in this article.

In fact, FDispEn considers the differences between adjacent elements of dispersion patterns, termed fluctuation-based dispersion patterns. In this way, we have vectors with length m−1, which each of their elements changes from −c+1 to c−1. Thus, there are ${\left(2c-1\right)}^{m-1}$ potential fluctuation-based dispersion patterns. The only difference between DispEn and FDispEn algorithms is the potential patterns used in these two approaches. Note that we use the normalized FDispEn as $\frac{\mathrm{FDispEn}}{\ln({\left(2c-1\right)}^{m-1})}$ herein.

As an example, let us have a signal x={3,4.5,6.2,5.1,3.2,1.2,3.5,5.6,4.9,8.4}. We set d=1, m=3, and c=2, leading to have $3^{2}=9$ potential fluctuation-based dispersion patterns ({(−1,−1),(−1,0),(−1,1),(0,−1),(0,0),(0,1),(1,−1),(1,0),(1,1)}). Then, $x_{j}$ (j=1,2,…,10) are linearly mapped into two classes with integer indices from 1 to 2 ({1,1,2,2,1,1,1,2,2,2}). Afterwards, a window with length 3 moves along the time series and the differences between adjacent elements are calculated ({(0,1),(1,0),(0,−1),(−1,0),(0,0),(0,1),(1,0),(0,0)}). Afterwards, the number of each fluctuation-based dispersion pattern is counted. Finally, using Equation (2), the DispEn value of **x** is equal to $-(\frac{1}{8}\ln\left(\frac{1}{8}\right)+\frac{1}{8}\ln\left(\frac{1}{8}\right)+\frac{2}{8}\ln\left(\frac{2}{8}\right)+\frac{2}{8}\ln\left(\frac{2}{8}\right)+\frac{2}{8}\ln\left(\frac{2}{8}\right))=1.5596$.

### 2.3. Mapping Approaches Used in DispEn and FDispEn

A number of linear and nonlinear methods can be used to map the original signal $x_{j}\left(j=1,2,\ldots ,N\right)$ to the classified signal $u_{j}\left(j=1,2,\ldots ,N\right)$. The simplest and fastest algorithm is the linear mapping. However, when maximum or minimum values are noticeably larger or smaller than the mean/median value of the signal, the majority of $x_{j}$ are mapped to only a few classes. To alleviate the problem, we can sort $x_{j}\left(j=1,2,\ldots ,N\right)$ and then divide them into *c* classes in which each of them includes equal number of $x_{j}$ (DispEn or FDispEn with sorting method).

We also use several nonlinear mapping techniques. Many natural processes show a progression from small beginnings that accelerates and approaches a climax over time (e.g., a sigmoid function) [28,29]. When there is not a detailed description, a sigmoid function is frequently used [29,30,31]. Well-known log-sigmoid (logsig) and tan-sigmoid (tansig) transfer functions are respectively defined as:

(3) $$y_{j}=\frac{1}{1+e^{-\frac{x_{j}-\mu }{\sigma }}},$$

(4) $$y_{j}=\frac{2}{1+e^{-2\frac{x_{j}-\mu }{\sigma }}}-1,$$

where σ and μ are the standard deviation (SD) and mean of time series **x**, respectively.

The cumulative distribution functions (CDFs) for many common probability distributions are sigmoidal. The most well-known such example is the error function, which is related to the CDF of a normal distribution, termed normal CDF (NCDF). NCDF of **x** is calculated as follows:

(5) $$y_{j}=\frac{1}{\sigma \sqrt{2\pi }}\int_{-\infty }^{x_{j}}e^{\frac{-{\left(t-\mu \right)}^{2}}{2\sigma^{2}}}\;dt.$$

Each of the aforementioned techniques maps x into $y=\{y_{1},y_{2},\ldots ,y_{N}\}$, ranged from α to β. Then, we use a linear algorithm to assign each $y_{j}$ to a real number $z_{j}$ from 0.5 to c+0.5. Next, for each element of the mapped signal, we use $u_{j}^{c}=\mathrm{round}\left(z_{j}\right)$, where $u_{j}^{c}$ denotes the *j*^th^ element of the classified signal and rounding involves either increasing or decreasing a number to the next digit [9]. It is worth noting that DispEn with NCDF and DispEn with linear mapping were compared by the use of several synthetic time series and four biomedical and mechanical datasets [9]. The results illustrated the superiority of DispEn with NCDF over DispEn with linear mapping.

## 3. Parameters of DispEn and FDispEn

### 3.1. Effect of Number of Classes, Embedding Dimension, and Signal Length on DispEn and FDispEn

**Figure 2:**
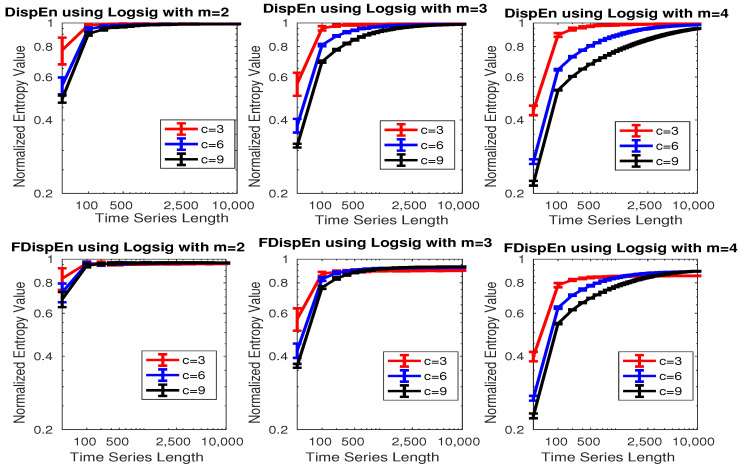
Mean and SD of results obtained by the DispEn and FDispEn with logsig and different values of embedding dimension and number of classes for 40 realizations of univariate white noise. Logarithm scale for both of the axes is used.

To assess the sensitivity of DispEn and FDispEn with logsig, and PerEn to the signal length, embedding dimension *m*, and number of classes *c*, we use 40 realizations of univariate white noise. Note that we will show why logsig is an appropriate mapping technique for DispEn and FDispEn to characterize signals. The mean and SD of results, depicted in Figure 2, show that DispEn and FDispEn need a smaller number of sample points to reach their maximum values for a smaller number of classes or smaller embedding dimension. This is in agreement with the fact that we need at least $\ln(c^{m})$ [9] and $\ln({\left(2c-1\right)}^{m-1})$ sample points to reach the maximum value of DispEn and FDispEn, respectively. The profiles also suggest that the greater the number of sample points, the more robust DispEn estimates, as seen from the errorbars.

### 3.2. Effect of Number of Classes and Noise Power on DispEn and FDispEn

We also inspect the relationship between noise power levels and DispEn with a different number of classes. To this end, we use a logistic map added with different levels of noise power. Signals created by biological systems are usually nonlinear and most likely include deterministic and stochastic components [13,32,33,34]. The reason why the logistic map is very popular in this field (e.g., [10,14,35,36]) is that its behavior changes from periodicity to non-periodic nonlinearity when α changes from 3.5 to 4 [37,38,39]. We then added white Gaussian noise (WGN) to the signal since real signals, especially physiological recordings, are frequently corrupted by different kinds of noise [40]. Additive WGN is also considered as a basic statistical model used in information theory to mimic the effect of random processes that occur in nature [41].

This analysis is dependent on the model parameter α as: $x_{j}=\alpha x_{j-1}\left(1-x_{j-1}\right)$, where the signal x was generated with the different values α (e.g., 3.5, 3.6, 3.7, 3.8, 3.9, and 4). The length and sampling frequency of the signal are, respectively, 500 sample points and 150 Hz. In case α equals 3.5, the time series oscillates among four values. For 3.57≤α≤4, the series is chaotic, albeit it has segments with periodic behaviour (e.g., α≈3.8) [38,39,42]. We added 40 independent realizations of WGN with different signal-to-noise-ratios (SNRs) per sample, ranging from 0 to 30 dB, to the logistic map.

To compare the sensitivity of each method to WGN, we calculate *NrmEntN* as the entropy value of each signal with noise over the entropy value of its corresponding signal without noise (NrmEntN=entropyofaserieswithnoiseentropyofaserieswithoutnoise).

**Figure 3:**
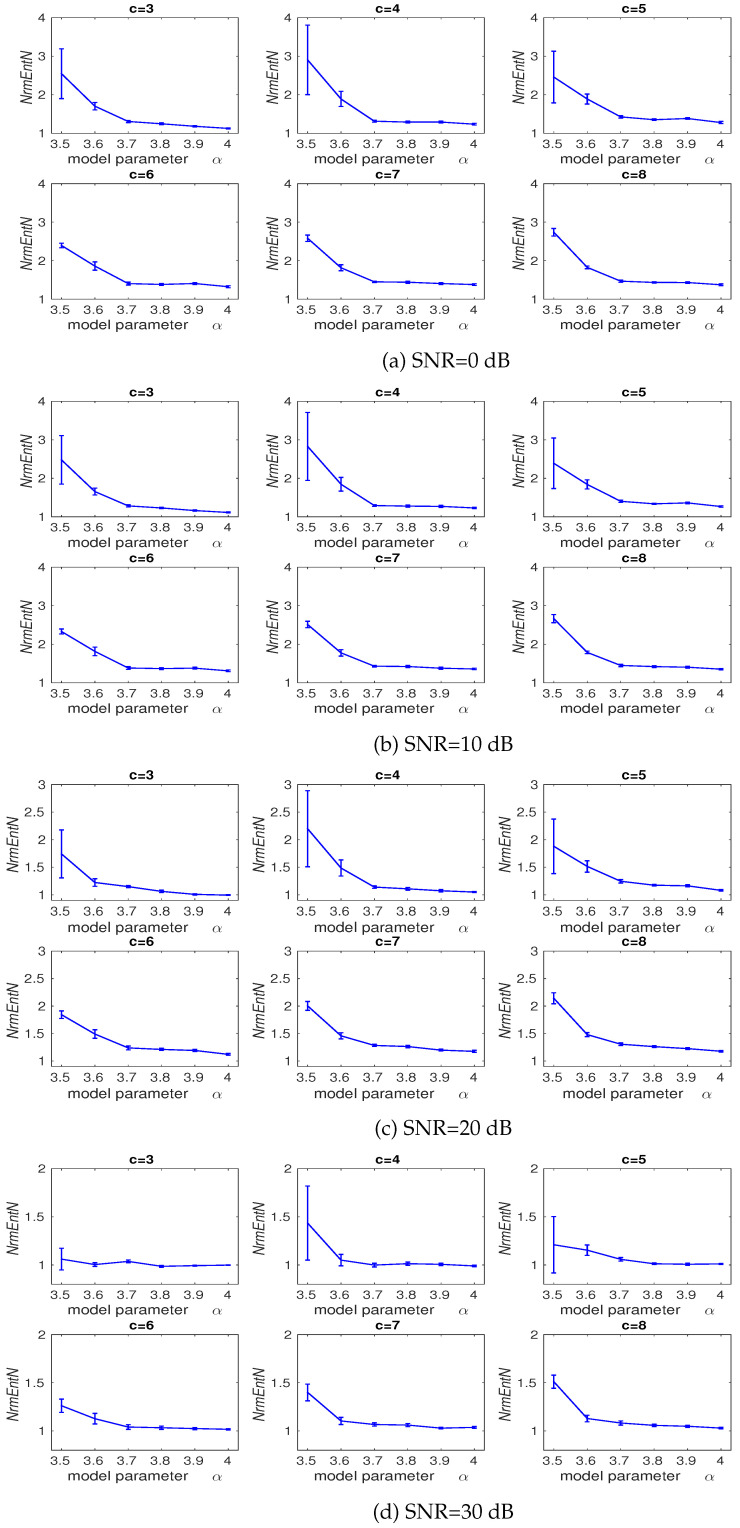
Average and SD of NrmEntN=entropyofaserieswithnoiseentropyofaserieswithoutnoise values obtained by the DispEn using logsig with a different number of classes computed from the logistic map with additive 40 independent realizations of WGNs with different noise power. *NrmEntN* compares the sensitivity of DispEn to WGN with different SNRs.

The average and SD values of results obtained by the DispEn using logsig with a different number of classes computed from the logistic map whose parameter (α) is equal to 3.5, 3.6, 3.7, 3.8, 3.9, or 4 with additive 40 independent realizations of WGN with SNR 0, 10, 20, 30 dB are shown in Figure 3a–d, respectively. We set m=2 for DispEn [9]. Figure 3 suggests that the SD values for c=6 are considerably smaller than those for c=5, 4, and 3. Moreover, the average of *NrmEntN* values for c=6 is smaller than those for c=7, and 8, showing less sensitivity to noise for c=6. Thus, we set c=6 for all the simulations below.

Compared with DispEn, in the FDispEn algorithm, we have vectors with length m−1 where each of their elements changes from −c+1 to c−1. Thus, we set m=3 here. Like what we did for DispEn, we changed *c* from 4 to 9 for FDispEn. We found that c=5 leads to stable results when dealing with noise (results are not shown herein). Thus, we set c=5 for all simulations using FDispEn, although the range 3<c<9 results in similar profiles.

Overall, the parameter *c* is chosen to balance the quantity of entropy estimates with the loss of signal information. To avoid the impact of noise on signals, a small *c* is recommended. In contrast, for a small *c*, too much detailed data information is lost, leading to poor probability estimates. Thus, a trade-off between large and small *c* values is needed.

## 4. Evaluation of Mapping Approaches for DispEn and FDispEn

**Figure 4:**
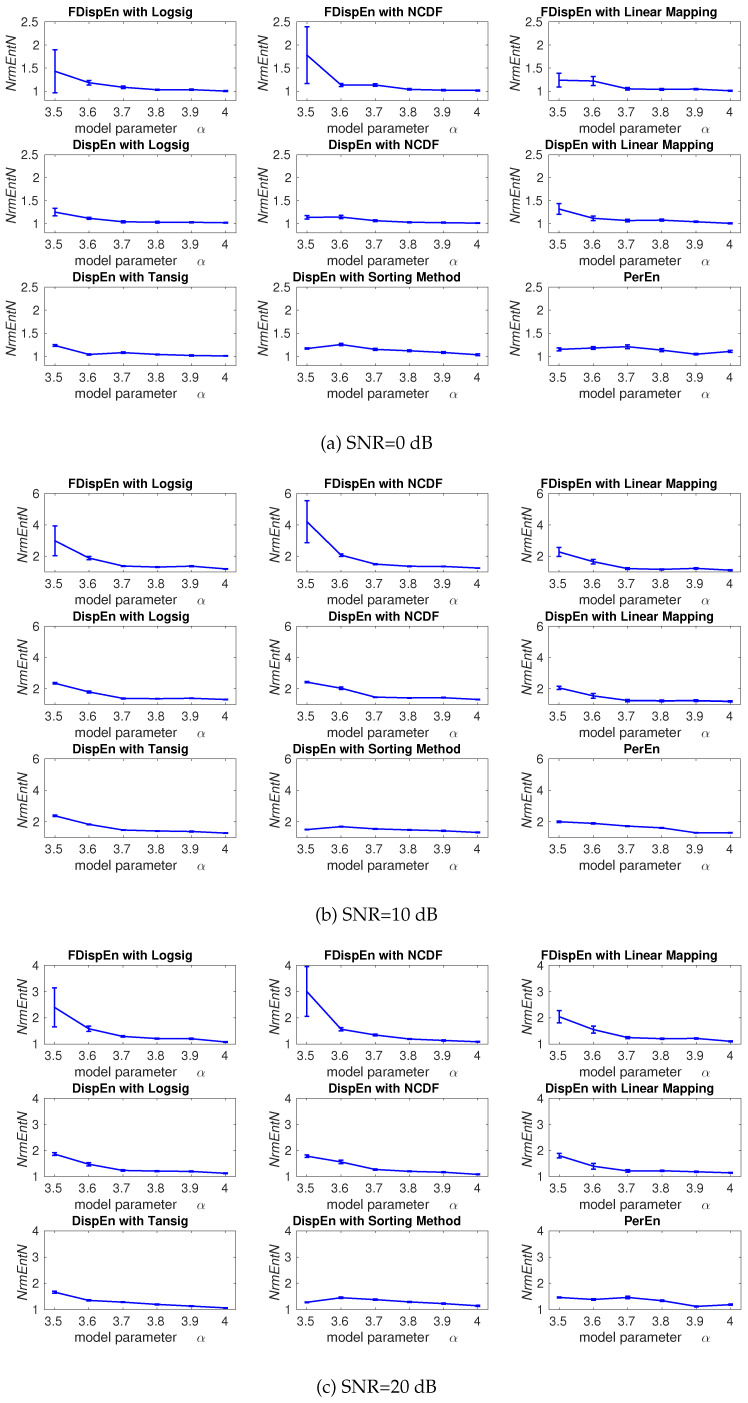

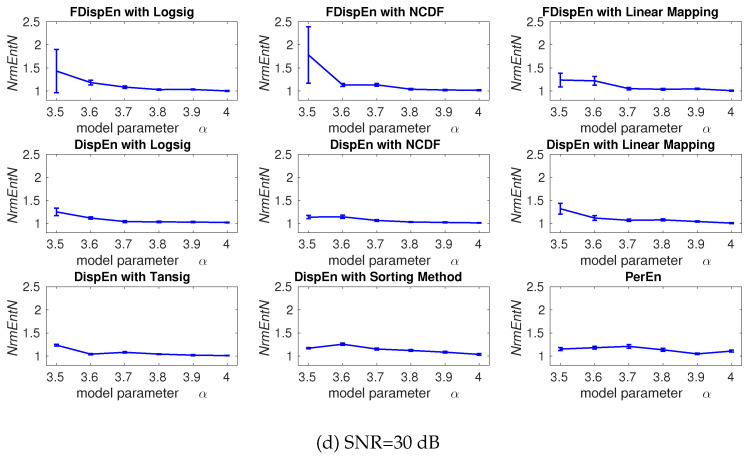
Average and SD of NrmEntN=entropyofaserieswithnoiseentropyofaserieswithoutnoise values obtained by the PerEn, and DispEn and FDispEn with different mapping techniques computed from the logistic map with additive 40 independent realizations of WGNs with different noise power. *NrmEntN* compares the sensitivity of each method to WGN with different SNRs.

To evaluate the ability of DispEn and FDispEn with different mapping techniques to distinguish changes from periodicity to non-periodic nonlinearity with different levels of noise, the described logistic map with additive noise is used. The average and SD of results obtained by the DispEn and FDispEn with different mapping techniques, and PerEn are depicted in Figure 4. The entropy values of the logistic map generally increase along the signal, except for the segments of periodic behavior (e.g., for α=3.8), in agreement with Figure 4.10 (page 87 in [39]) and previous studies [42,43]. We set m=2 and m=3 for DispEn and FDispEn, respectively.

As noise affects more in periodic oscillations, *NrmEntN* is larger for a small α. The range of mean values show that DispEn and FDispEn with different mapping algorithms, and PerEn are similar, while dealing with the different levels of noise power. The SD values suggest that when all signals have equal SNR values, the DispEn and PerEn values are stable for all the methods.

The ranges of mean values show that DispEn with sorting method and linear mapping lead to the most stable results. Although DispEn with sorting method, unlike PerEn, takes into account repetitions, it considers only the order of amplitude values and, thus, some information regarding the amplitudes may be discarded. For instance, DispEn with sorting method cannot detect the outliers or spikes, which is noticeably larger or smaller than their adjacent values. For DispEn with linear mapping, when maximum or minimum values are noticeably larger or smaller than the mean/median value of the signal, the majority of $x_{j}$ are mapped to only a few classes [9]. Thus, for simplicity, we use DispEn and FDispEn with logsig for all the simulations below.

Noise is frequently considered as an unwanted component or disturbance to a system or data, whereas recent studies have shown that noise can play a beneficial role in systems [44,45]. In any case, it has been made evident that noise is an essential ingredient in the systems and has a noticeable effect on many aspects of science and technology, such as engineering, medicine, and biology [44,45]. White, pink, and brown noise are three well-known kinds of noise signals in the real world. White noise is a random signal having equal energy across all frequencies. The power spectral density of white noise is as $S\left(f\right)=C_{w}$, where $C_{w}$ is a constant [45]. Pink and brown noise are random processes suitable for modelling evolutionary or developmental systems [46]. The power spectral density S(f) of pink and brown noise are as $\frac{C_{p}}{f}$ and $\frac{C_{b}}{f^{2}}$, respectively, where $C_{p}$ and $C_{b}$ are constants [45,46].

To evaluate the ability of DispEn and FDispEn methods with different mapping algorithms, and PerEn to distinguish the dynamics of different noise signals, we created 40 realizations of white, brown, and pink noise signals with different lengths changing from 10 to 1000 sample points. Note that, as the maximum value of PerEn is ln(m!) [47], we use normalized PerEn as $\frac{\mathrm{PerEn}}{\ln(m!)}$ in this study. We set m=4 for PerEn [48], m=2 and c=6 for DispEn [9], and m=3 and c=5 for FDispEn as recommended before.

**Figure 5:**
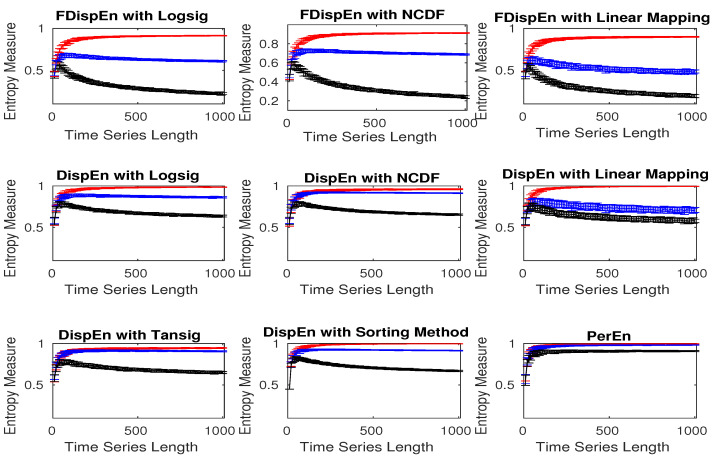
Mean and SD of entropy values obtained by DispEn and FDispEn with different mapping techniques and PerEn, computed from 40 different white noise (red colour), pink noise (blue colour), and brown noise (black colour).

Figure 5 shows that DispEn and FDispEn with different mapping approaches distinguish brown, pink, and white noise series with different lengths. Their results are in agreement with the fact that white noise is the most irregular signal, followed by pink and brown noise, in that order, based on the power spectral density of white, pink, and brown noise [44,45]. However, there are some overlaps between the DispEn with tansig, and PerEn values for short pink and white noise time series, suggesting a superiority of DispEn and FDispEn with different mapping approaches, except tansig, over PerEn.

## 5. Univariate Entropy Methods vs. Changes from Periodicity to Non-Periodic Nonlinearity

**Figure 6:**
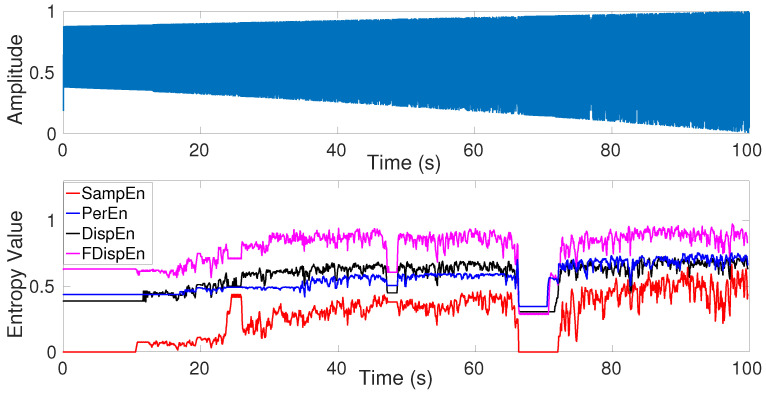
Logistic map with parameter α changing from 3.5 to 3.99 and entropy values of the logistic map to understand better SampEn, PerEn, DispEn, and FDispEn.

Studies on physiological time series frequently involve relatively short epochs of signals containing informative periodic or quasi-periodic components [13,49,50]. Moreover, empirical evidence identifies nonlinear, in addition to linear, behavior in some biomedical signals [32,51,52]. Therefore, to find the dependence of univariate entropy approaches with changes from periodicity to non-periodic nonlinearity, a logistic map is used herein. This analysis is relevant to the model parameter α as: $x_{j}=\alpha x_{j-1}\left(1-x_{j-1}\right)$, where the signal $x=x_{j}\;\left(j=1,\ldots ,N\right)$ was generated varying the parameter α from 3.5 to 3.99. We employed a sliding window of 60 sample points with 80% overlap moves along the signal with a sampling frequency of 150 Hz and a length of 100 s (15,000 sample points). The signal is depicted in Figure 6. We set m=2 for SampEn, DispEn, and FDispEn, and m=3 for PerEn, as advised before.

The results obtained by FDispEn, DispEn, PerEn, and SampEn for the logistic map are shown in Figure 6. For each of the methods, when 3.5<α<3.57 (periodic series), the entropy values are smaller than those for 3.57<α<3.99 (chaotic series), except those epochs that include periodic components (e.g., α≈3.8) [38,39,42]. As expected, the entropy values, obtained by the entropy techniques generally increase along the signal, except for the downward spikes in the windows of periodic behavior (α≈3.8). This fact is in agreement with Figure 4.10 (page 87 in [39]) and the other previous studies [10,16].

## 6. Comparison Between SampEn, PerEn and Its Improvements, and Newly Developed DispEn and FDispEn

In this section, we compare the DispEn and FDispEn algorithms with the SampEn and PerEn-based methods.

### 6.1. SampEn vs. DispEn and FDispEn

In addition, DispEn, FDispEn, and SampEn have similar behavior when dealing with noise. In SampEn, only the number of matches whose differences are smaller than a defined threshold is counted. Accordingly, a small change in the signal amplitude due to noise is unlikely to modify the SampEn value. Similarly, in DispEn and FDispEn, a small change will probably not alter the index of class and so the entropy value will not change. Therefore, SampEn, DispEn, and FDispEn are relatively robust to noise (especially for signals with high SNR).

The relationship between the number of classes *c* (DispEn and FDispEn) and threshold *r* (SampEn) is inspected by the use of a MIX process evolving from randomness to periodic oscillations as follows [35,42]:

(6) $$MIX_{k}=\left(1-z_{k}\right)x_{k}+z_{k}y_{k},$$

where $z=\{z_{1},z_{2},\ldots ,z_{N}\}$ is a random variable that is equal to 1 with probability *p* and equal to 0 with probability 1−p, $x=\{x_{1},x_{2},\ldots ,x_{N}\}$ denotes a periodic synthetic time series created by $x_{k}=\sqrt{2}\sin\left(\frac{2\pi k}{12}\right)$, and $y=\{y_{1},y_{2},\ldots ,y_{N}\}$ is a uniformly distributed variable on $\left[-\sqrt{3},\sqrt{3}\right]$ [35,42]. The time series was based on a MIX process whose parameter linearly varied between 0.99 and 0.01. Therefore, this series evolved from randomness to orderliness. The signal has a sampling frequency of 150 Hz and a length of 100 s (15,000 samples). The techniques are applied to 20 realizations of the MIX process using a moving window of 1500 samples (10 s) with 50% overlap. We used different threshold values r=0.1,0.2,0.3,0.4, and 0.5 of SD of the signal [14] for SampEn, and c=2,4,6,8 and 10 for DispEn and FDispEn.

**Figure 7:**
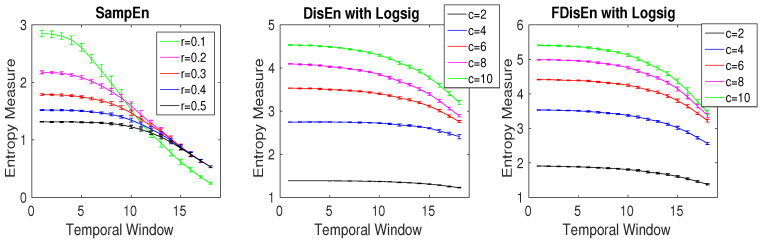
Average and SD of entropy values obtained by the DispEn, FDispEn, and SampEn with different numbers of classes (for DispEn and FDispEn) and different threshold values (SampEn) using a MIX process evolving from randomness to periodic oscillations. We used a window with length 1500 samples moving along the MIX process (temporal window).

The results, depicted in Figure 7, show that the mean entropy values are the lowest in higher temporal windows, in agreement with the previous studies [35,42]. The results also show that the number of classes (*c*) in DispEn and FDispEn is inversely related to the threshold value *r* used in the SampEn algorithm. It is worth noting that SampEn, unlike DispEn and FDispEn, is not consistent as r=0.1 crosses the lines for other values of *r*. We set m=2, 2, and 3, for, respectively, SampEn, DispEn, and FDispEn, as recommended before.

To compare the results obtained by the entropy algorithms, we used the coefficient of variation (CV) defined as the SD divided by the mean. We use such a metric as the SDs of signals may increase or decrease proportionally to the mean. We inspect the MIX process with length 1500 samples and p=0.5 as a trade-off between random (p=1) and periodic oscillations (p=0). The CV values, depicted in Table 1, show that DispEn- and FDispEn results for different number of classes are noticeably smaller than those for SampEn with different threshold values, showing another advantage of DispEn and FDispEn over SampEn.

**Figure 8:**
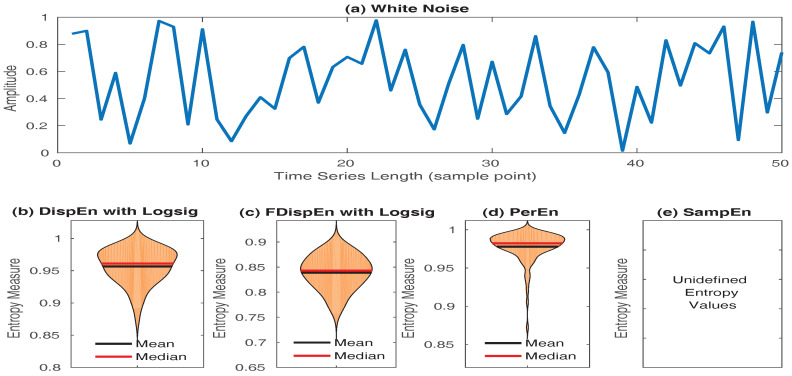
Mean and median of results obtained by (**b**) DispEn; (**c**) FDispEn with logsig; (**d**) PerEn and (**e**) SampEn, for 40 realizations of (**a**) white noise.

In spite of its power to detect dynamics of signals, SampEn has two key deficiencies. They are discussed as follows:

1. SampEn values for short signals are either undefined or unreliable, as in its algorithm, the number of matches whose differences are smaller than a defined threshold is counted. When the time series length is too small, this number may be 0, leading to undefined values [16,53]. However, the results obtained by DispEn, FDispEn, and PerEn are always defined. To illustrate this issue, we created 40 realizations of white noise with length 50 sample points. The mean and median of DispEn, FDispEn, PerEn, and SampEn values for the 40 realizations are shown in Figure 8. The results show that SampEn, unlike DispEn, FDispEn, and PerEn, yield undefined values. Note that we set m=2 for SampEn, DispEn, and FDispEn, and m=3 for PerEn, as advised before.
2. SampEn is not fast enough for real time applications and has a computation cost of O($N^{2}$) [54]. In contrast, the computation cost of PerEn, DispEn, and FDispEn is O(*N*) [9,55].

### 6.2. PerEn and Its Improvements vs. DispEn and FDispEn

PerEn, DispEn, and FDispEn are based on the Shannon’s definition of entropy, reflecting the average uncertainty of a random variable [11,12]. Nevertheless, these techniques have the following main differences:

1. PerEn considers only the order of amplitude values, and, thus, some information regarding the amplitude values themselves may be ignored [18]. For example, the embedded vectors {1,10,2} and {1,3,2} have similar permutations, leading to the same motif (0,2,1) (m=3) because the extent of the differences between sequential samples is not considered in the original definition of PerEn. To alleviate this deficiency, modified PerEn (MPerEn) based on mapping equal values into the same symbol was developed [17]. However, the second and third shortcomings were not addressed by MPerEn. Amplitude-aware PerEn (AAPerEn) deals with the problem with adding a variable contribution, depending on amplitude, instead of a constant number to each level in the histogram representing the probability of each motif [7]. It was also addressed by the use of modified ordinal patterns [56]. Mapping data to a number of classes based on their amplitude values makes DispEn and FDispEn deal with this issue as well.
2. When there are equal values in the embedded vector, Bandt and Pompe [10] proposed ranking the possible equalities based on their order of emergence or solving this condition by adding noise. Considering the first alternative, for instance, the permutation pattern for both the embedded vectors {1,2,4} and {1,4,4} are (0,1,2) (m=3). As another example, assume z1={1,2,2,2} and z2={1,2,3,4}. The PerEn with m=3 of z1 is exactly the same as z2, both equalling 0 although, unlike z1, z2 is strictly ascending. Adding noise may not lead to a precise answer because, for example, the embedded vector {1,5,5} has two possible permutation patterns as (0,1,2) and (0,2,1) and there are not any differences between them. It should be noted that this issue is particularly relevant for digitized signals with large quantization steps. Fadlallah et al. have recently proposed weighted PerEn (WPerEn) to weight the motif counts by statistics derived from the time series patterns [8]. However, WPerEn does not take into account the first and third alleviations of PerEn. It was addressed in AAPerEn [7] as well. Assigning close amplitude values to an equal class, FDispEn and DispEn deal with this deficiency.
3. PerEn is sensitive to noise (even when the SNR of a signal is high), since a small change in amplitude value may vary the order relations among amplitudes. For instance, noise on z3={1,2,2.01} may alter the motif from (0,1,2) to (0,2,1). This problem is present for WPerEn, MPerEn, AAPerEn, and the approach developed in [56]. However, DispEn and FDispEn address the problem with mapping data into a few classes and, thus, a small change in amplitude will probably not alter the (index of) class.

**Figure 9:**
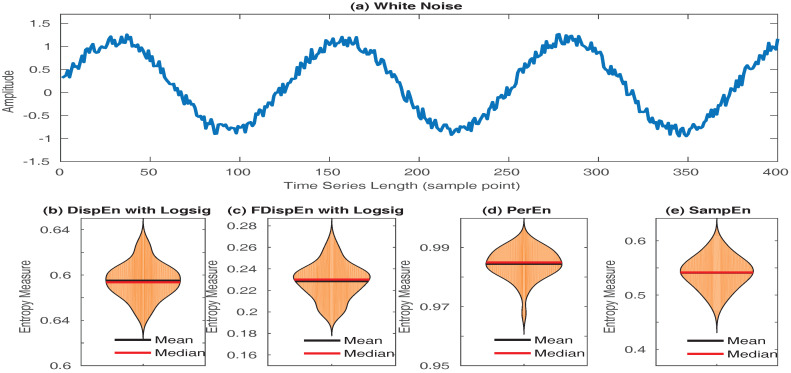
Mean and median of results obtained by (**b**) DispEn; (**c**) FDispEn with logsig; (**d**) PerEn and (**e**) SampEn, for 20 realizations of (**a**) $x_{i}=\sin\left(i/20\right)+0.3\eta$.

To demonstrate this issue, let us have twenty realizations of the signal $x_{i}=\sin\left(i/20\right)+0.3\eta$ with length 400 sample points, where η denotes a uniform random variable between 0 to 1. The original signal, and the mean and median of DispEn, FDispEn, PerEn, and SampEn values for the twenty time series are depicted in Figure 9. The results show that the mean PerEn of these realizations is close to the PerEn of a random signal (i.e., both are close to 1). In contrast, for the other entropy methods, there is a considerable difference between the entropy values and their corresponding maximum entropy. Of note is that we set m=3 for DispEn and FDispEn, m=2 for SampEn, and m=4 for PerEn.

To summarize, the characteristics and limitations of DispEn [9], FDispEn, SampEn [14], AAPerEn [7], and PerEn [10] are illustrated in Table 2.

## 7. Computation Cost of DispEn, FDispEn, and PerEn

In order to assess the computational time of DispEn and FDispEn with logsig, compared with PerEn, we use random time series with different lengths, changing from 300 to 100,000 sample points. The results are depicted in Table 3. The simulations have been carried out using a PC with Intel (R) Xeon (R) CPU, E5420, 2.5 GHz and 8 GB RAM by MATLAB R2015a. The number of classes for FDispEn and DispEn was 6. Additionally, DispEn and FDispEn with logsig were used for all the simulations.

The results show that the computation times of SampEn with different *m* are very close, while for DispEn, FDispEn, and PerEn, the larger the *m* value, the higher the computation time. PerEn is the fastest algorithm. For long signals and m=2, 3, and 4, FDispEn is relatively faster than DispEn. For a long time series, the running times of SampEn are considerably higher than those for DispEn, FDispEn, and PerEn. This is in agreement with the fact that the computation costs of DispEn, FDispEn, PerEn, and SampEn are, respectively, O(*N*), O(*N*), O(*N*), and O($N^{2}$) [9,54]. Of note is that the optimised implementation of PerEn was used in this article [56], whereas the straightforward implementations of DispEn and FDispEn were utilized.

## 8. Forbidden Amplitude- and Fluctuation-Based Dispersion Patterns

In this section, we introduce forbidden amplitude- and fluctuation-based dispersion patterns and explore the use of these concepts to discriminate deterministic from stochastic time series. Forbidden patterns denote those patterns that do not appear at all in the analysed signal [18,57]. There are two reasons behind the existence of forbidden patterns. First, a signal with finite length does not have a number of potential patterns (false forbidden patterns). For example, the time series {1,2,3,2.1,1,4} has only four permutations from six potential permutation patterns with m=3. Thus, the permutations {231} and {312} can be considered as false forbidden patterns. The second reason is based on the dynamical nature of the systems creating a signal. When signals made by an unconstrained stochastic process, all possible permutation patterns appear and there is no forbidden pattern. In contrast, it was made evident that deterministic one-dimensional maps always have forbidden permutation or ordinal patterns [57,58].

Based on a null hypothesis, we illustrate that it is impossible that, for the embedding dimension *m*, we have all the dispersion patterns, but not all the permutation patterns.

- Step 1: Null hypothesis. We have all the dispersion patterns, while the permutation pattern $\left(\ell_{1},\ell_{2},\ldots ,\ell_{m}\right)$ does not exist for the signal **x**.
- Step 2: Rejection of null hypothesis. As the permutation pattern $\left(\ell_{1},\ell_{2},\ldots ,\ell_{m}\right)$ does not exist, we do not have any dispersion patterns sorted as $\left(\ell_{1},\ell_{2},\ldots ,\ell_{m}\right)$. This is in contradiction with the fact that we have all the dispersion patterns for **x**. Hence, the null hypothesis is rejected.
- Step 3: Conclusion. When we have all the dispersion patterns, all the permutation patterns are present too. It confirms the fact that a forbidden permutation pattern leads to several forbidden dispersion patterns. Thus, if a signal is deterministic, and so does not have several permutation patterns, there are a number of forbidden dispersion patterns. Consequently, lack of dispersion patterns, like permutation patterns [57,58], reflects the deterministic behavior of a signal.

Conversely, when there is a forbidden dispersion pattern or fluctuation-based dispersion pattern for a signal, the time series is not stochastic. Thus, there is at least one forbidden permutation pattern as well. It is worth noting that the null hypothesis for FDispEn is similar.

To illustrate this issue, an example is provided: we set m=3 for DispEn, FDispEn and PerEn and c=6 for DispEn and FDispEn. If the permutation pattern (2,3,1) does not exist for the signal **x**, we do not have the following dispersion patterns: (2,3,1), (2,4,1), (2,5,1), (2,6,1), (3,4,1), (3,5,1), (3,6,1), (4,5,1), (4,6,1), (5,6,1), (3,4,2), (3,5,2), (3,6,2), (4,5,2), (4,6,2), (5,6,2), (4,5,3), (4,6,3), (5,6,3), and (5,6,4); and fluctuation-based dispersion patterns: (1,−2), (2,−3), (3,−4), (4,−5), (1,−3), (2,−4), (3,−5), (1,−4), (2,−5), (1,−5), (1,−2), (2,−3), (3,−4), (1,−3), (2,−4), (1,−4), (1,−2), (2,−3), (1,−3), and (1,−2). This demonstrates that lack of a permutation pattern results in lack of several dispersion and fluctuation-based dispersion patterns. Accordingly, as permutation patterns are used to discriminate deterministic from stochastic series based on lack of permutation patterns [57,58], dispersion and fluctuation-based patterns are able to be utilized as well.

**Figure 10:**
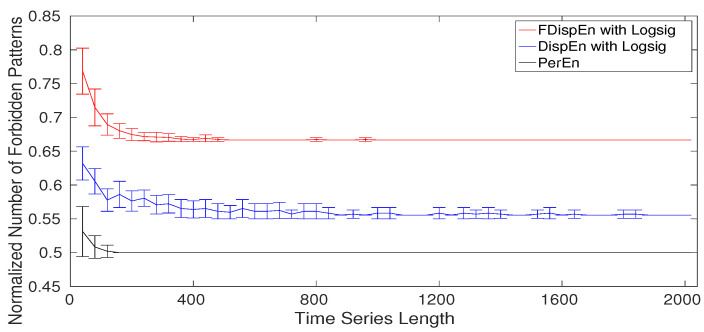
Mean and SD of the normalized number of forbidden amplitude- and fluctuation-based dispersion and permutation patterns (numberofforbiddenpatternspotentialnumberofpatterns) as functions of the signal length.

The normalized number of forbidden (missing) dispersion and permutation patterns as a function of the signal length using the logistic map $x_{t+1}=4x_{t}\left(1-x_{t}\right)$ [58] for DispEn and FDispEn with logsig, and PerEn are shown in Figure 10. Note that the normalized number of forbidden patterns is equal to the number of forbidden patterns over the potential number of patterns (m!, $c^{m}$, and ${\left(2c-1\right)}^{m-1}$ for, respectively, PerEn, DispEn, and FDispEn). As can be seen in Figure 10, for short signals, we have a number of false forbidden patterns. The results make evident that more than half of the dispersion and permutation patterns are forbidden. On the whole, the results show that both the amplitude- and fluctuation-based dispersion patterns can be used to differentiate deterministic from stochastic time series.

## 9. Applications of DispEn and FDispEn to Biomedical Time Series

Physiologists and clinicians are often confronted with the problem of distinguishing different kinds of dynamics of biomedical signals, such as heart rate tracings from infants who had an aborted sudden infant death syndrome versus control infants [32], and electroencephalogram (EEG) signals from young versus elderly people [59]. A number of physiological time series, such as cardiovascular, blood pressure, and brain activity recordings, show a nonlinear in addition to linear behaviour [60,61,62]. Moreover, several studies suggested that physiological recordings from healthy subjects have nonlinear complex relationships with ageing and disease [13]. Thus, there is an increasing interest in nonlinear techniques, especially entropy-based metrics, to analyse the dynamics of physiological signals. To this end, to evaluate the DispEn and FDispEn methods to quantify the degree of the uncertainty of biomedical signals, we use two publicly-available datasets from http://www.physionet.org. The proposed methods are compared with PerEn, Lempel–Ziv complexity (LZC), and SampEn.

### 9.1. Blood Pressure in Rats

We evaluate the ability of entropy methods and LZC on the non-invasive blood pressure signals from nine salt-sensitive hypertensive (SS) Dahl rats and six rats protected (SP) from high-salt-induced hypertension (SSBN13) on a high-salt diet (8% salt) for two weeks [34,63]. Each blood pressure signal was recorded using radiotelemetry for two minutes with sampling frequency of 100 Hz. The study was approved by the Institutional Animal Care and Use Committee of the Medical College of Wisconsin-Madison, US [34,63]. Further information can be found in [34,63].

**Figure 11:**
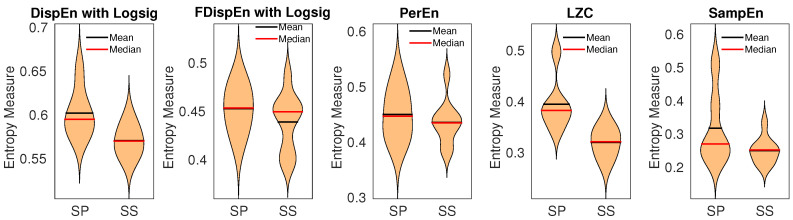
Mean and median of results obtained by PerEn, LZC, SampEn, and DispEn and FDispEn with logsig from salt-sensitive (SS) vs. salt protected (SP) rats’ blood pressure signals.

As the entropy approaches are used for stationary signals [10,14], we separated each signal into epochs with length 4 s (400 sample points) and applied the methods to each of them. Next, the average entropy value of all the epochs was calculated for each signal. The results, illustrated in Figure 11, show a loss of uncertainty with the salt-sensitive rats, in agreement with [63]. We set m=4 for PerEn [48], m=2 and r=0.2 multiplied by SD of each epoch for SampEn, and m=3 for both DispEn and FDispEn. The Hedges’ g effect size [64] was employed to assess the differences between results for SS versus SSBN13 Dahl rats. The differences, illustrated in Table 4, show that the best algorithm to discriminate the SS from SSBN13 Dahl rats is LZC, followed by DispEn, SampEn, FDispEn, and PerEn, in that order.

### 9.2. Gait Maturation Database

We also used the gait maturation database to assess the entropy methods to distinguish the effect of age on the intrinsic stride-to-stride dynamics [65]. A subset including 23 healthy boys and girls is considered in this study. The children were classified into two age groups: 3 and 4 years old (11 subjects) and 11 to 14 years old children (12 subjects). Height and weight of the young and elderly groups were 105±2 cm and 155±10 cm, and 17.3±0.7 kg, and 44.4±2.7 kg, respectively. The time series recorded from the subjects walking at their normal pace have the lengths of about 400–500 sample points. For more information, please see [65].

**Figure 12:**
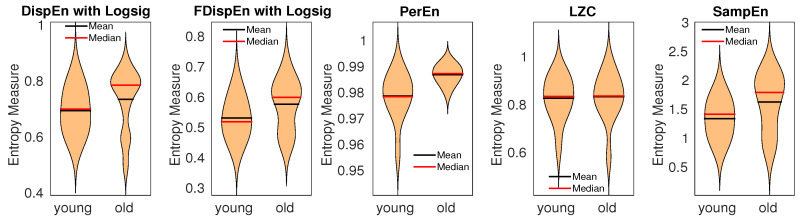
Mean and median of results obtained by PerEn, LZC, SampEn, and DispEn and FDispEn with logsig for young and elderly children’s stride-to-stride recordings.

The results, depicted in Figure 12, show that the average entropy values obtained by DispEn and FDispEn with logsig, SampEn, and PerEn for the elderly children are larger than those for the young children, in agreement with previous studies [66,67]. The parameters values for the entropy methods are equal to those used for the blood pressure in rats. The differences for the elderly vs. young children based on Hedges’ g effect size are shown in Table 4. The results demonstrate that DispEn, FDispEn, and SampEn outperform PerEn and LZC to distinguish various dynamics of the stride-to-stride recordings.

Overall, the results for the two real datasets demonstrate an advantage of DispEn and FDispEn with logsig over PerEn to distinguish different types of dynamics of the biomedical recordings. However, we acknowledge that there may be other datasets where PerEn outperforms DispEn and FDispEn. In any case, our results show the potential of DispEn and FDispEn for characterization of biomedical signals. Furthermore, the differences for the blood pressure and gait maturation datasets are shown that DispEn is the most consistent algorithm to distinguish the dynamics of signals for the real datasets. In spite of the promising findings and results for different applications aforementioned in this pilot study, further investigations into potential applications of DispEn and FDispEn are recommended.

## 10. Conclusions

In this paper, we carried out an investigation aimed at gaining a better understanding of our recently developed DispEn, especially regarding the parameters and mapping techniques used in DispEn. We also introduced FDispEn to quantify the uncertainty of time series in this article. The basis of this technique lies in taking into account only the local fluctuations of signals. The concepts of forbidden amplitude- and fluctuation-based dispersion patterns were also introduced in this study.

The work done here has the following implications for uncertainty or irregularity estimation. Firstly, we showed that DispEn and FDispEn with logsig are appropriate approaches when dealing with noise. We also found that the forbidden amplitude- and fluctuation-based dispersion patterns are suitable to distinguish deterministic from stochastic time series. Additionally, the results showed that both DispEn and FDispEn with logsig distinguish various physiological states of the two biomedical time series better than PerEn. Finally, the most consistent method to distinguish the different states of physiological signals was DispEn with logsig, compared with FDispEn with logsig, LZC, PerEn, and SampEn.

Due to their low computational cost and ability to detect dynamics of signals, we hope DispEn and FDispEn can be used for the analysis of a wide range of physiological and even non-physiological signals.

## Figures and Tables

**Table 1:** CVs of DispEn and FDispEn with logsig, and SampEn values for the MIX process with p=0.5 and length 1000 samples.

Method	c=2	c=4	c=6	c=8	c=10
DispEn	0.0021	0.0034	0.0045	0.0041	0.0048
	c=2	c=4	c=6	c=8	c=10
FDispEn	0.0078	0.0064	0.0040	0.0043	0.0049
	r=0.1× SD	r=0.2× SD	r=0.3× SD	r=0.4× SD	r=0.5× SD
SampEn	0.0604	0.0342	0.0224	0.0174	0.0150

**Table 2:** Comparison between DispEn and FDispEn and SampEn, PerEn, and AAPerEn in terms of ability to characterize short signals, sensitivity to noise, type of entropy, and computational cost.

Characteristics	DispEn	FDispEn	AAPerEn	PerEn	SampEn
Short signals	reliable	reliable	reliable	reliable	undefined
Sensitivity to noise	no	no	yes	yes	no
Type of entropy	ShEn	ShEn	ShEn	ShEn	ConEn
Computational cost	O(*N*)	O(*N*)	O(*N*)	O(*N*)	O($N^{2}$)

**Table 3:** Computational time of DispEn and FDispEn with logsig, SampEn, and PerEn with different embedding dimension values and signal lengths.

Number of Samples	300	1000	3000	10,000	30,000	100,000
DispEn (m=2)	0.0022 s	0.0022 s	0.0025 s	0.0057 s	0.0080 s	0.0225 s
DispEn (m=3)	0.0028 s	0.0035 s	0.0076 s	0.0115 s	0.0284 s	0.0888 s
DispEn (m=4)	0.0084 s	0.0094 s	0.0205 s	0.0505 s	0.1422 s	0.4752 s
FDispEn (m=2)	0.0022 s	0.0025 s	0.0028 s	0.0034 s	0.0062 s	0.0175 s
FDispEn (m=3)	0.0025 s	0.0031 s	0.0038 s	0.0062 s	0.0150 s	0.0490 s
FDispEn (m=4)	0.0054 s	0.0064 s	0.0120 s	0.0284 s	0.0699 s	0.2535 s
SampEn (m=2)	0.0023 s	0.0208 s	0.1841 s	1.8478 s	16.8394 s	193.1970 s
SampEn (m=3)	0.0022 s	0.0206 s	0.1808 s	1.8337 s	16.9200 s	189.4041 s
SampEn (m=4)	0.0019 s	0.0193 s	0.1631 s	1.8322 s	16.5596 s	189.1037 s
PerEn (m=2)	0.0014 s	0.0015 s	0.0016 s	0.0020 s	0.0034 s	0.0099 s
PerEn (m=3)	0.0014 s	0.0016 s	0.0016 s	0.0024 s	0.0043 s	0.0115 s
PerEn (m=4)	0.0015 s	0.0016 s	0.0019 s	0.0026 s	0.0054 s	0.0113 s

**Table 4:** Differences between results for SS vs. SSBN13 Dahl rats (blood pressure data), and for elderly vs. young children (gait maturation dataset) obtained by DispEn and FDispEn with logsig, LZC, SampEn, and PerEn based on the Hedges’ g effect size.

Dataset	DispEn	FDispEn	PerEn	LZC	SampEn
Blood pressure	1.35 (very large)	0.46 (medium)	0.31 (small)	1.74 (huge)	0.84 (large)
Gait maturation	0.74 (large)	0.75 (large)	0.63 (medium)	0.16 (small)	0.79 (large)
